# Regulatory Effects of IFN-β on the Development of Experimental Autoimmune Uveoretinitis in B10RIII Mice

**DOI:** 10.1371/journal.pone.0019870

**Published:** 2011-05-06

**Authors:** Min Sun, Yan Yang, Peizeng Yang, Bo Lei, Liping Du, Aize Kijlstra

**Affiliations:** 1 The First Affiliated Hospital of Chongqing Medical University, Chongqing Key Laboratory of Ophthalmology and Chongqing Eye Institute, Chongqing, People's Republic of China; 2 Zhongshan Ophthalmic Center, Sun Yat-sen University, Guangzhou, People's Republic of China; 3 Eye Research Institute Maastricht, Department of Ophthalmology, University Hospital Maastricht, Maastricht, The Netherlands; Institute Biomedical Research August Pi Sunyer (IDIBAPS) - Hospital Clinic of Barcelona, Spain

## Abstract

**Background:**

Experimental autoimmune uveoretinitis (EAU) serves as a model for human intraocular inflammation. IFN-β has been used in the treatment of certain autoimmune diseases. Earlier studies showed that it ameliorated EAU; however, the mechanisms involved in this inhibition are still largely unknown.

**Methodology/Principal Findings:**

B10RIII mice were immunized with interphotoreceptor retinoid-binding protein (IRBP) peptide 161–180 in Complete Freund's adjuvant. Splenocytes from different time points after immunization were used to evaluate the expression of IFN-β. An increased expression of IFN-β was observed during EAU and its highest expression was observed on day 16, 3 days after the peak of intraocular inflammation. Splenocytes and draining lymph node cells from mice immunized with IRBP_161-180_ on day 13 and control mice were activated with anti-CD3/anti-CD28 antibodies or IRBP_161-180_ to evaluate the production of IFN-γ and IL-17. The results showed that IFN-γ and IL-17 were significantly higher in immunized mice as compared to the control mice when exposed to anti-CD3/anti-CD28 antibodies. However, the production of IFN-γ and IL-17 was detected only in immunized mice, but not in the control mice when stimulated with IRBP_161-180_. Multiple subcutaneous injections of IFN-β significantly inhibited EAU activity in association with a down-regulated expression of IFN-γ, IL-17 and an enhanced IL-10 production. In an in vitro system using cells from mice, IFN-β suppressed IFN-γ production by CD4^+^CD62L^−^ T cells, IL-17 production by CD4^+^CD62L^+/-^ T cells and proliferation of CD4^+^CD62L^+/-^ T cells. IFN-β inhibited the secretion of IL-6, but promoted the secretion of IL-10 by monocytes. IFN-β-treated monocytes inhibited IL-17 secretion by CD4^+^CD62L^+/-^ T cells, but did not influence IFN-γ expression and T cell proliferation.

**Conclusions/Significance:**

IFN-β may exert its inhibitory effect on EAU by inhibiting Th1, Th17 cells and modulating relevant cytokines. IFN-β may provide a potential treatment for diseases mediated by Th1 and Th17 cells.

## Introduction

Interferon (IFN)-β, belongs to the type I interferons, and is secreted by fibroblasts, monocytes-macrophages and dendritic cells (DC) during the innate immune response against viral pathogens [1]. It has been shown to be effective in the treatment of relapsing-remitting multiple sclerosis [2]–[3] and various studies have revealed that it could inhibit inflammation through regulating T helper (Th) 1 and Th2 cytokines [4]–[6]. Additional evidence indicates that IFN-β promotes the production of interleukin (IL)-10 and inhibits pro-inflammatory cytokines [7]–[9].

Naive CD4^+^ T cells differentiate into distinct subsets after activation by professional antigen-presenting cells (APC). Traditionally, CD4^+^ effector T cells have been classified into two subsets: Th1 and Th2 lineages [10]. Th1 cells are induced by IL-12 and produce large amounts of IFN-γ, whereas Th2 cells predominantly secrete IL-4, IL-5 and IL-13. Th1 cells orchestrate cellular immunity and Th2 cells regulate humoral immunity and allergic responses. Recently, Th17 cells have been added to these lineages and have now been well characterized both in humans and animals. Th17 cells have been shown to be involved in the pathogenesis of various clinical autoimmune diseases as well as their experimental counterparts in animals [11]–[14]. It has been shown that the differentiation of naïve T cells into Th17 cells is dependent on transforming growth factor (TGF)-β and IL-6, whereas the maintenance and expansion of Th17 cells is sustained by IL-23 [12], [15]–[17].

Experimental autoimmune uveoretinitis (EAU) is an organ specific autoimmune disease. It has been induced in a variety of animals such as rat and mouse by immunization with a number of antigens including interphotoreceptor retinoid-binding protein (IRBP), the soluble retinal antigen [18] or peptides derived from these autoantigens. Clinical and histopathological studies have shown a high similarity between this model and human uveitis. Therefore, this model has been widely used as a counterpart for human intraocular inflammation [19]. Early studies have shown that EAU was mainly mediated by Th1 cells. Th2 cells may provide a protection against this model. Recent studies have shown that a new Th subset that produces IL-17 (Th17) is also involved in the development of EAU [20]–[21]. As mentioned above, the inhibitory role of IFN-β has been reported in MS patients and in animal models of autoimmune encephalomyelitis [2], [22]–[24]. IFN-α, which also belongs to the type I interferons, has been used in Europe to treat various forms of uveitis [25]–[27] and EAU [28]–[29]. Earlier studies from Japan have shown that oral IFN-β could ameliorate EAU in rats and evidence was obtained that this was mediated via NK or NKT cells [30]. Whether IFN-β may exert its effect by interfering with Th1 and/or Th17 lineages was not addressed yet and was therefore the subject of the study presented here. Our in vivo experiments revealed that IFN-β could significantly ameliorate the severity of EAU in association with an inhibition of Th1, Th17 cells and an enhanced expression of IL-10. In vitro experiments showed that it inhibited the production of pro-inflammatory cytokines including IFN-γ, IL-17 and IL-6, but promoted the production of IL-10. It also suppressed the proliferation of naïve T cells and effector/memory T cells. Additionally, IFN-β-treated monocytes were able to inhibit the production of IL-17 by naïve T cells and effector/memory T cells.

## Results

### Induction of EAU and the dynamic expression of IFN-β in this model

**Figure 1:**
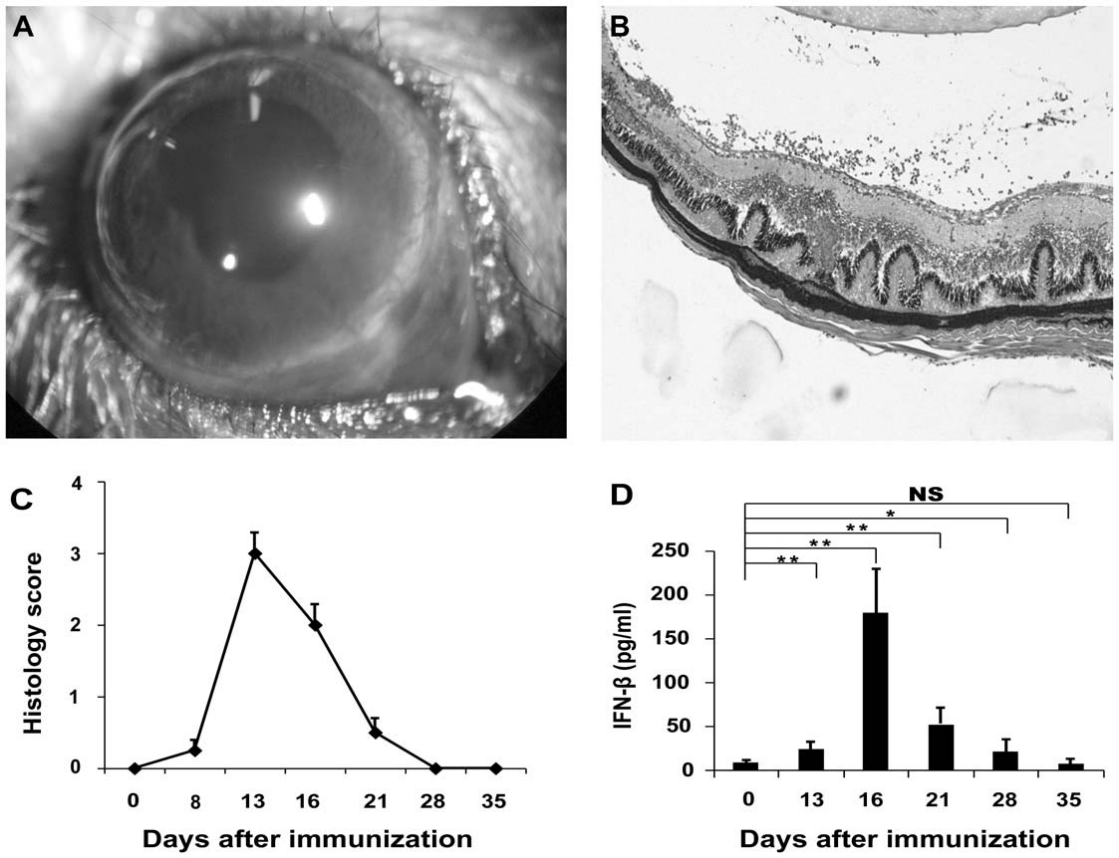
Induction of EAU and analysis of the expression of IFN-β in this model. EAU was induced in B10RIII mice by immunization with 50 µg IRBP_161-180_ in CFA. (A) Inflammatory infiltration in the anterior segment of the eye (after mydriasis). (B) Damaged retinal structure in a B10RIII mouse after immunization with IRBP_161-180_. Eyes were obtained 13 days after immunization and stained with haematoxylin/eosin. (C) Mean histology score during the development of EAU. (D) Splenocytes from normal mice (day 0) and those obtained at different time points after the induction of EAU were stimulated by LPS for 24 hours. Supernatants were measured for the production of IFN-β by ELISA. Data are representative of three independent experiments. **p<0.01, *p<0.05, NS = not significant.

EAU was successfully induced in B10RIII mice and was characterized clinically by aqueous flare, cells, and exudates in the anterior chamber. Histological examination showed a severe inflammation in the anterior segment as well as in the posterior segment as evidenced by a massive influx of mononuclear and polymorphonuclear cells into the retina and choroid, granuloma formation, vasculitis, photoreceptor loss and vitritis (Figure 1A, B). This inflammation became evident on day 8 or 9 and reached its peak on day 13, followed by a rapid regression (Figure 1C). No intraocular inflammation was observed in the control mice.

Splenocytes and lymph node cells from normal mice and those at different time points following immunization with IRBP_161-180_ were stimulated with LPS and the supernatants were assessed for IFN-β expression. Lymph node cells did not express IFN-β, whereas IFN-β expression in splenocytes was significantly higher on day 13 and reached its peak on day 16, followed by a gradual decrease and returning to normal levels on day 35 (Figure 1D).

**Figure 2:**
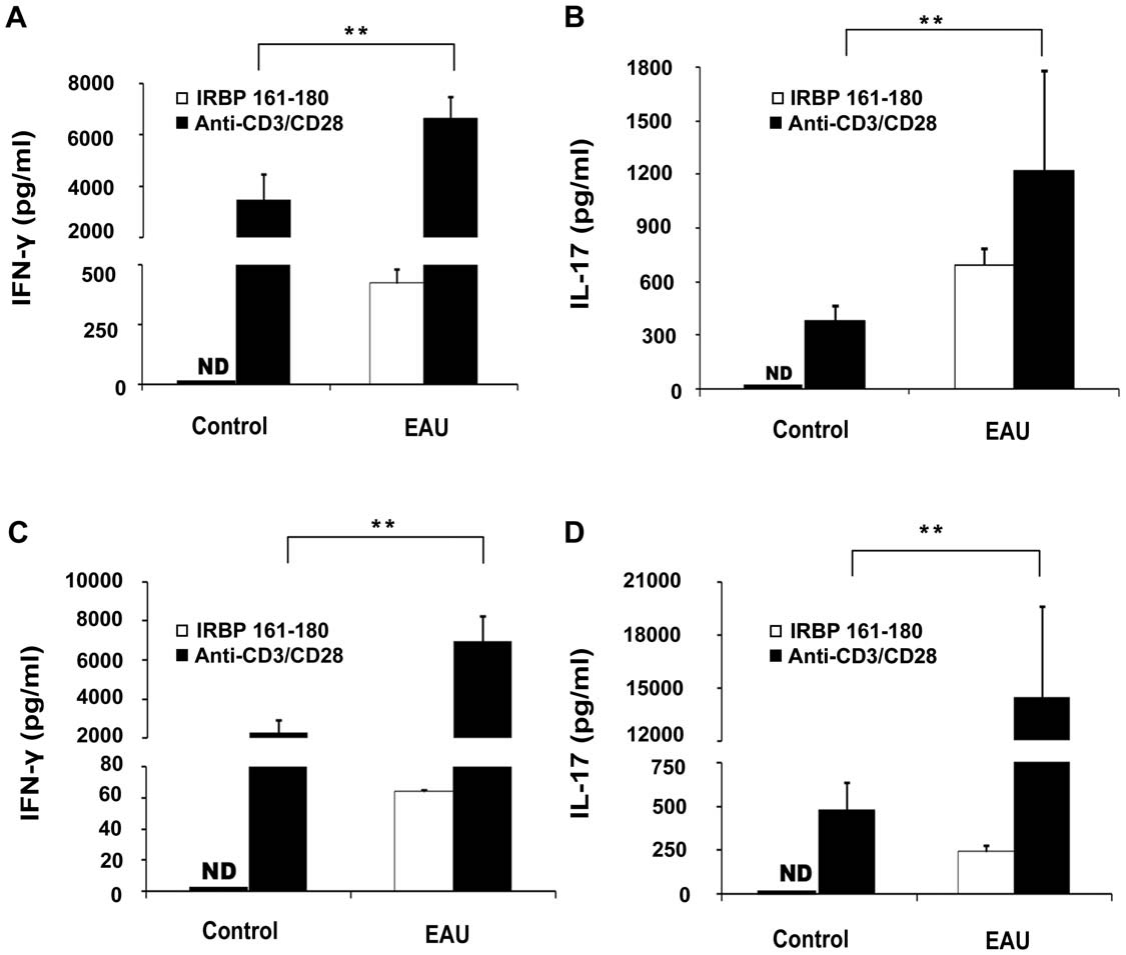
The expression of IFN-γ and IL-17 in EAU mice and control mice. Splenocytes (A and B) and DLN cells (C and D), obtained from EAU mice or control mice on day 13, were activated with IRBP_161-180_ (20 µg/ml) or anti-CD3 and anti-CD28 (1 µg/ml) for 72 hours. IFN-γ and IL-17 were analyzed by ELISA. Data are representative of three independent experiments. **p<0.01, ND =  not detected.

IFN-γ and IL-17 production by splenocytes and draining lymph node (DLN) cells were detected by enzyme-linked immunosorbent assay (ELISA) on day 13 after immunization. The results showed that IFN-γ and IL-17 in the supernatants of splenocytes and DLN cells stimulated with anti-CD3 and anti-CD28 antibodies from the immunized mice were significantly higher than those from the control mice. The production of IFN-γ and IL-17 was detected in the immunized mice, but not in the control mice when stimulated with IRBP_161-180_. However, their production was significantly lower in the experiment with IRBP_161-180_ stimulation than that with anti-CD3 and anti-CD28 antibodies stimulation (Figure 2).

### *In vivo* influence of IFN-β on EAU activity, production of cytokines and T cell proliferation

**Figure 3:**
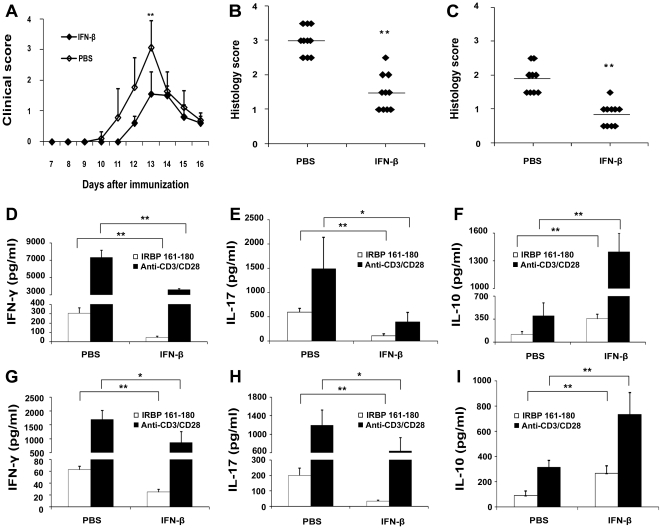
The influence of IFN-β treatment on EAU activity, production of IFN- γ, IL-17 and IL-10. Clinical scores (A) and histology scores on day 13(B) and day 16 (C) of IFN-β or PBS treated EAU mice. Splenocytes (D-F) and DLN cells (G-I) from day 16 with or without IFN-β treatment were activated with IRBP_161-180_ (20 µg/ml) or anti-CD3 and anti-CD28 (1 µg/ml) for 72 hours. Cytokines were analyzed by ELISA. Data are representative of three independent experiments with at least five mice per group. **p<0.01, *p<0.05.

IFN-β was administered to mice before and after immunization with IRBP_161-180_ to examine its influence on EAU activity and various other parameters. Clinical examination showed that IFN-β significantly inhibited EAU activity on day 13 (Fig. 3A). However, it did not influence the incidence or duration of EAU. Histopathological examination on day 13 or 16 following immunization revealed that the EAU score in IFN-β-treated mice was significantly lower than that found in control mice (Figure 3B, C).

As the highest expression of IFN-β was observed in the splenocytes on day 16, the splenocytes and DLN cells obtained from this time point were used to evaluate the influence of IFN-β on the production of several cytokines and T cell proliferation. The results showed that the production of IFN-γ and IL-17 by splenocytes or DLN cells was significantly decreased in IFN-β-treated mice as compared with the control mice. However, the levels of IL-10 in the supernatants of splenocytes and DLN cells were significantly higher in IFN-β-treated mice as compared with controls (Figure 3D–I). IFN-β did not influence the T cell proliferation in the spleen or in the DLN (data not shown).

### *In vitro* effect of IFN-β on cytokine production by Th1, Th17 and monocytes and T cell proliferation

**Figure 4:**
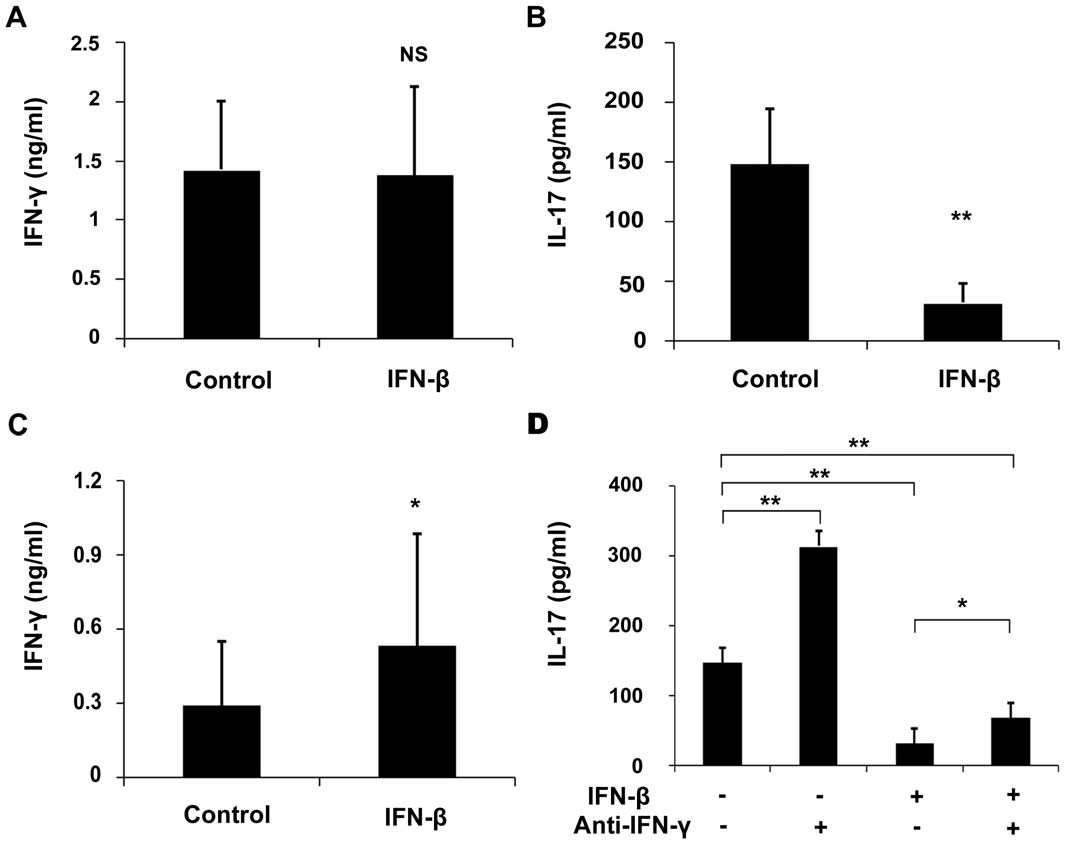
Effect of IFN-β on Th1 and Th17 differentiation. Naïve T cells cultured with or without IFN-β in Th1-polarizing conditions (10 ng/ml IL-12) (A) , Th17-polarizing conditions (20 ng/ml IL-6, 5 ng/ml TGF-β1 and 10 ng/ml IL-23) (B-C) or (20 ng/ml IL-6, 5 ng/ml TGF-β1 and 10 ng/ml IL-23 with or without 2 µg/ml anti-IFN-γ) (D) for 96 hours. IFN-γ and IL-17 were analyzed by ELISA. Data are representative of three independent experiments. **p<0.01, *p<0.05, NS = not significant.

An experiment was designed to examine whether IFN-β could directly inhibit the polarization of Th1 and Th17 cells. CD4^+^CD62L^+^ (naïve) T cells from normal mice stimulated with IL-12 were cultured with or without IFN-β to evaluate its influence on the production of IFN-γ. The results showed that it did not affect the production of IFN-γ (Figure 4A). CD4^+^CD62L^+^ T cells stimulated with TGF-β1, IL-6 and IL-23 were cultured with or without IFN-β to evaluate its effect on the production of IFN-γ and IL-17. The result showed that it significantly inhibited the production of IL-17 (Figure 4B), whereas it promoted the IFN-γ secretion by these T cells (Figure 4C). An additional experiment using anti-IFN-γ antibody was used to examine whether the inhibition of IFN-β on IL-17 was mediated by the up-regulated IFN-γ. The result showed that neutralization by anti-IFN-γ antibody resulted in a significantly increased IL-17 expression under Th17 polarization conditions (Figure 4D), suggesting that it could partially reverse the inhibition of IFN-β on IL-17 production.

**Figure 5:**
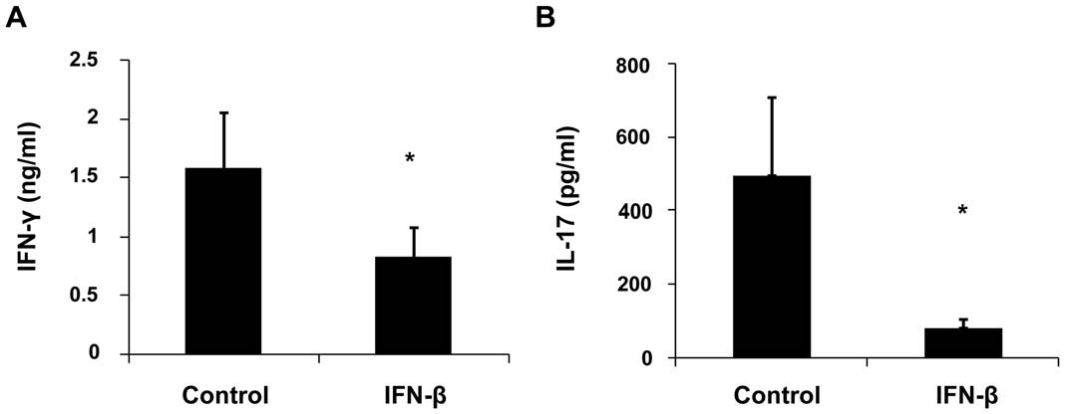
Effect of IFN-β on the expression of IFN-γ and IL-17 by effector/memory T cells. Effector/memory T cells were cultured with or without IFN-β for 72 hours. Cytokines were detected by ELISA. Data are representative of three independent experiments. *p<0.05.

CD4^+^CD62L^−^ T cells (effector/memory) from normal mice were cultured with anti-CD3 and anti-CD28 antibodies in the presence or absence of IFN-β to examine its influence on the production of cytokines by these cells. The results showed that IFN-β significantly inhibited the production of both IFN-γ and IL-17 (Figure 5).

**Figure 6:**
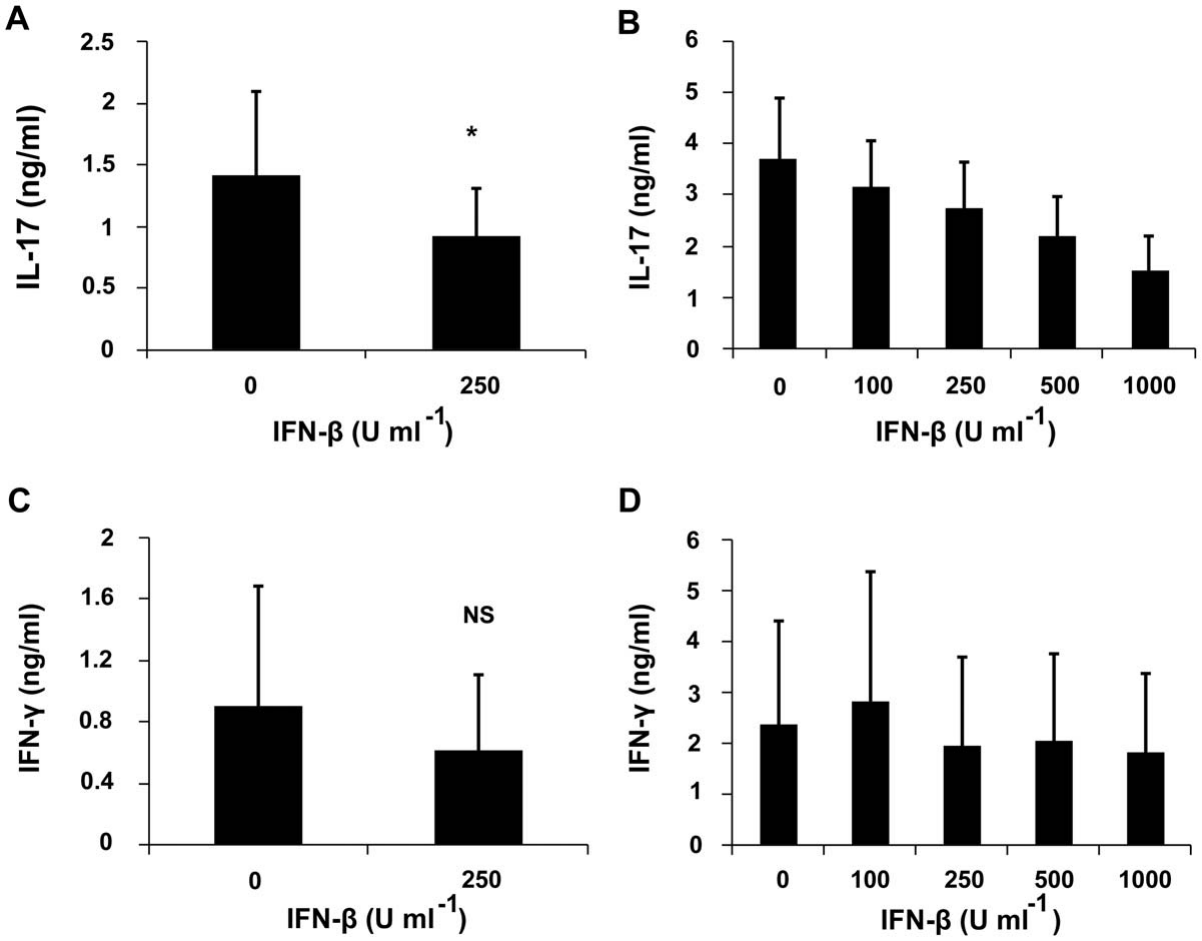
Effect of IFN-β-treated monocytes on the expression of IFN-γ and IL-17. Naïve T cells (A and B) and effector/memory T cells (C and D) were cultured with IFN-β-treated monocytes at a ratio of 5:1 for 72 hours. Cytokines were detected by ELISA. Data are representative of three independent experiments. *p<0.05, NS = not significant.

The aforementioned experiment revealed a direct inhibition of IFN-β on the production of IFN-γ and IL-17. As the receptor of IFN-β is also expressed by monocytes [31], a further study was performed to test whether IFN-β could also exert its effects on IFN-γ and IL-17 production through monocytes. The results showed that IFN-β-treated monocytes significantly inhibited the production of IL-17 by both CD4^+^CD62L^+^ T cells and CD4^+^CD62L^−^ T cells (Figure 6A, B). Furthermore, the inhibition of IFN-β-treated monocytes on CD4^+^CD62L^−^ T cells was dependent on IFN-β in a dose dependent manner. However, IFN-β-treated monocytes did not have any effect on IFN-γ secretion (Figure 6C, D).

**Figure 7:**
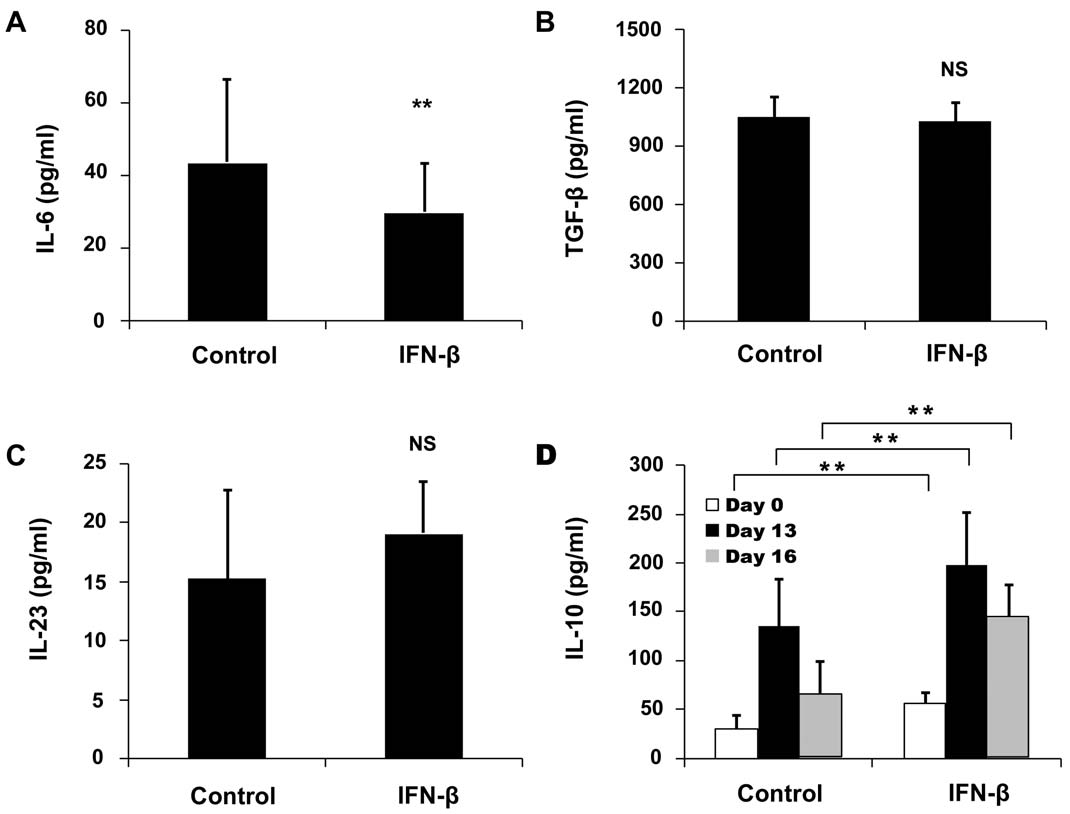
Effect of IFN-β on IL-6, TGF-β, IL-23 and IL-10 production by monocytes. Monocytes from immunized mice on day 13 (or on day 0 and day 16 where indicated), cultured with or without IFN-β (250 U/ml) for 24 hours, were stimulated with LPS (100 ng/ml) for another 24 hours. Cytokines were detected by ELISA. Data are representative of three independent experiments. **p<0.01, NS = not significant.

Monocytes from the mice immunized with IRBP_161-180_ on day 13 were cultured with or without IFN-β to evaluate its influence on the production of IL-6 and TGF-β, which are critical for the polarization of Th17 cells, and IL-23, which sustains the maintenance and expansion of Th17 cells. The results showed that it significantly inhibited the IL-6 production by these cells (Figure 7A). However, it did not have a detectable effect on the secretion of TGF-β and IL-23 by these cells (Figure 7B, C).

As IL-10 has anti-inflammatory properties and is involved in the regression of EAU [32], a further study was performed to detect whether IFN-β could exert its inhibitory role through up-regulating this cytokine. Our results showed that it significantly induced the monocytes from the normal mice and those immunized with IRBP_161-180_ on day 13 and 16 to abundantly produce IL-10. There was no difference concerning the ability of IFN-β to promote IL-10 production among the tested three groups (Figure 7D).

**Figure 8:**
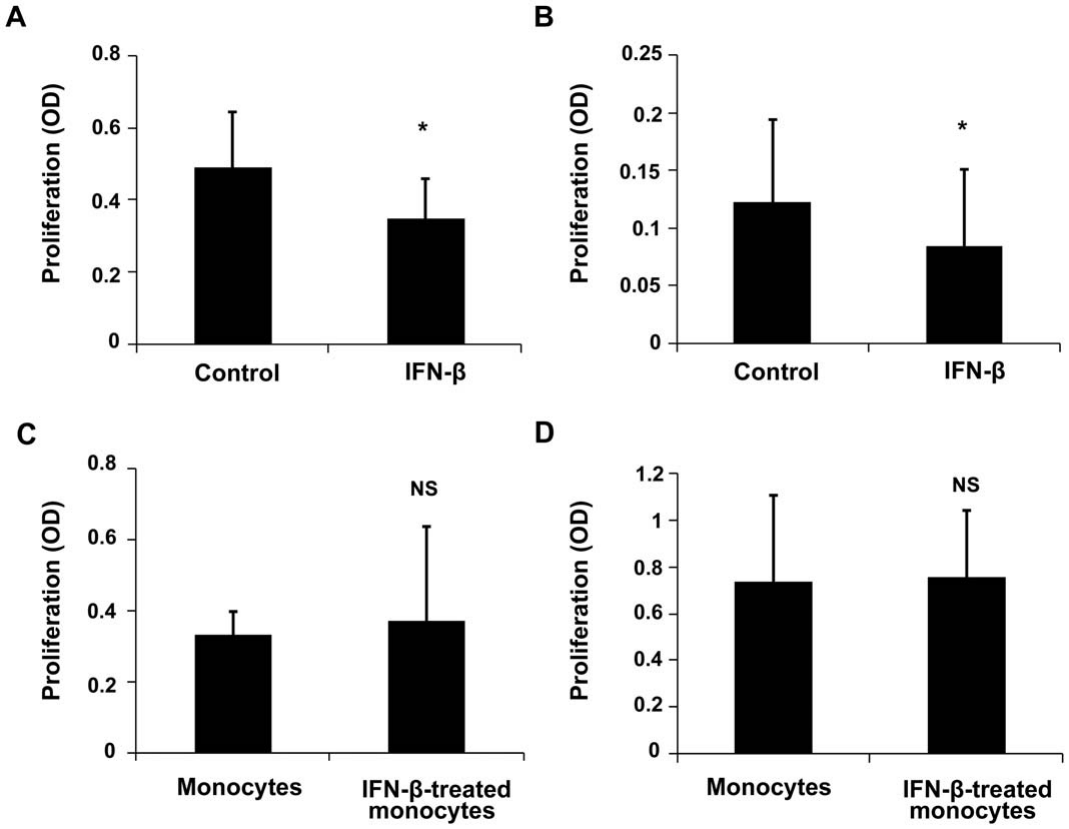
Effect of IFN-β on T cell proliferation. Naïve T cells (A) or effector/memory T cells (B) from normal mice cultured with anti-CD3 (1 µg/ml) and anti-CD28 (1 µg/ml) in the presence or absence IFN-β for 72 hours. IFN-β-treated monocytes cultured with naïve T cells (C) or effector/memory T cells (D) at a ratio of 1:5 for 72 hours. Proliferation was detected by modified MTT. Data are representative of three independent experiments with at least three mice per group. *p<0.05, NS = not significant.

Additional experiments were designed to examine whether IFN-β could directly inhibit the proliferation of CD4^+^CD62L^+^ T cells and CD4^+^CD62L^−^ T cells from normal mice. T cell proliferation was induced with anti-CD3 (1 µg/ml) and anti-CD28 (1 µg/ml) antibodies in the presence or absence of IFN-β and was then determined using a non radioactive colorimetric assay. IFN-β significantly inhibited the proliferation of both T cell subsets (Figure 8A, B). A further study was performed to investigate whether IFN-β could also exert its inhibitory effect on T cell proliferation through monocytes. CD4^+^CD62L^+^ T cells and CD4^+^CD62L^−^ T cells were cultured with or without IFN-β-treated monocytes and proliferation was measured as described above. IFN-β-treatment of monocytes did not influence the proliferation of CD4^+^CD62L^+^ T cells or CD4^+^CD62L^−^ T cells (Figure 8C, D).

## Discussion

In this study, we investigated the regulatory effect of IFN-β on EAU induced by immunization with IRBP_161-180_ and possible mechanisms involved in this process. We found an increased expression of IFN-β in association with an up-regulation of IFN-γ and IL-17 upon stimulation either with anti-CD3 and anti-CD28 antibodies or with IRBP_161-180_ during the active phase of inflammation. In vivo experiments showed that IFN-β significantly attenuated the EAU activity in association with a marked down-regulation of IFN-γ and IL-17. On the other hand, IFN-β treatment of mice undergoing EAU resulted in an almost three fold up-regulation of IL-10 production.

The anti-inflammatory and beneficial effect of type I interferons such as IFN-α and IFN-β in autoimmune diseases such as multiple sclerosis and uveitis [2], [3], [25]–[27] contrasts with a pro-inflammatory role for IFN-α in systemic lupus erythematosus and dermatomyositis [33]-[34]. Why type I interferons can act as pathogenic mediators in some diseases and on the other hand offer possibilities as a therapeutic agent in others is not clear yet.

Previous studies showed that IFN-α suppressed EAU by down-regulating TNF-α production [28] and reducing ocular CD45^+^ T cells infiltration [29]. Only few studies have addressed the effect of IFN-β on EAU and mainly concentrated on T cell proliferation and IFN-γ production [30]. We extended these studies and firstly examined whether and how IFN-β was expressed during this model. The highest expression of IFN-β was observed 3 days after the peak of inflammation which suggests that it may be involved in the resolution of EAU. IFN-β was mainly produced by splenocytes and not by draining lymph node cells which confirms earlier findings [35]. Further in vitro experiments showed that IFN-β significantly inhibited T cell proliferation as well as the production of IFN-γ, IL-17 and IL-6, whereas it markedly enhanced the production of IL-10. Collectively, these results suggest that IFN-β is able to inhibit EAU activity and that this inhibition is possibly mediated through modulating Th1, Th17 cells and IL-10 production. The second aim of our study was to investigate whether injection of IFN-β could influence the clinical activity of EAU as well as other relevant parameters. Our result showed that multiple injections of this protein before and after immunization with IRBP peptides could significantly ameliorate the EAU activity although it did not completely prevent the development of this disease. We did not investigate IFN-β administration at the time of disease onset since the model we used is characterized by a rapid self limiting regression of the disease and an eventual effect of IFN-β would probably not be detected. Further investigations concerning other time and dosage schemes are necessary to delineate the optimal conditions for the IFN-β effect on EAU. IFN-β significantly inhibited the production of IFN-γ and IL-17 upon nonspecific stimulation or specific antigen stimulation. On the other hand, it promoted the secretion of IL-10. Our data on IFN-γ production are in agreement with earlier studies by Suzuki et al. in the EAU model in rats [30]. These results are also consistent with those reported in the treatment of MS patients with IFN-β [2], [7], [36]. However, unexpectedly, we failed to find any influence of in vivo IFN-β treatment on T cell proliferation assays in vitro. This result is in disagreement with the data published by Suzuki et al. [30] but generally consistent with that reported by Hedegaard et al. [36]. In the latter study, IFN-β treatment of MS patients was not associated with an effect of this protein on T cell proliferation.

The third aim of our study was to examine how IFN-β exerted its inhibitory role using a number of in vitro experiments. The studies of the effects of IFN-β on the cytokine production of Th1 and Th17 cells showed that it significantly inhibited both cytokine production and polarization of Th17 cells, but only suppressed the cytokine production of Th1 cells. These results are generally in line with those reported by Axtell et al. [37]. They cultured APC with naïve T cells or effector/memory T cells in the presence of TGF-β and IL-6 and found that IFN-β significantly inhibited IL-17 production by both populations. We disclosed a significant inhibition of IFN-β on the production of IFN-γ by effector/memory T cells. Our results with regard to the effect of IFN-β on IFN-γ secretion by naïve T cells are consistent with those reported by Axtell et al. [37]. They did not find an effect of IFN-β by CD4^+^ T cells when these T cells were cultured with APC in the presence of IL-12. However, McRae et al. [38] found that IFN-β significantly inhibited the production of IFN-γ by naïve T cells when cultured with human DC. This discrepancy with regard to the IFN-β effect on IFN-γ between human and mice is not fully understood. The origin of IFN-β used in the experiments might be another reason to explain the observed difference.

Previous studies had shown that enhancing the Th2 response would be beneficial in clinical treatment of uveitis by preventing IFN-γ production [39]. IFN-β was able to boost the Th2 response in MS patients [6]. Whether IFN-β inhibits IFN-γ partially through enhancing Th2 responses is a matter of future studies.

IL-12 and IL-18, secreted by monocytes, are critical for Th1 proliferation and IFN-γ production by differentiated Th1 cells [40]. Our results showed that IFN-β did not influence IFN-γ production through monocytes in vitro. Whether IFN-β inhibits IFN-γ in vivo by down-regulating IL-12 and IL-18 production needs further clarification.

The aforementioned results indicated a direct inhibitory effect of IFN-β on the production of IL-17. Our further study showed that its inhibition on IL-17 could be mediated by indirect mechanisms. The experiment using anti-IFN-γ antibody revealed that the inhibitory role of IFN-β could also be mediated through enhancing the production of IFN-γ. The experiment using IFN-β-treated monocytes disclosed that IFN-β exerted its effect on IL-17 production through inhibiting IL-6 production by monocytes. It has been shown that IFN-β may also exert its effect on Th17 cells through modulating expression of IL-1β, IL-23 and IL-27 in both humans and mice [23], [41]-[42]. Further study is needed to clarify whether IFN-β could inhibit Th17 cells through these cytokines and whether it mediates its inhibitory effect on EAU via this pathway. It is also worthwhile to point out that we failed to find a detectable effect of IFN-β on the production of TGF-β, an important cytokine for Th17 polarization, and IL-23 by monocytes, a critical cytokine for survival of Th17 cells. Taken together, these results suggest that IFN-β not only inhibited the IL-17 production by direct or indirect routes, but also prevented Th17 polarization through modulating IL-6 secretion.

Our study also investigated the effects of IFN-β on IL-10 and T cell proliferation, which are all important factors in the immune response or in inflammation. The results showed that IFN-β significantly promoted the production of IL-10 by monocytes upon stimulation with LPS. This result is, by and large, in agreement with that reported by Chang et al. [43] They found that the effect of LPS stimulation on the production of IL-10 was principally mediated by IFN-β. The T cell proliferation experiment showed that IFN-β could significantly inhibit the proliferation of these cells, which is contrary to our in vivo findings but in agreement with earlier studies in rats with EAU [30].

In conclusion, our study showed that IFN-β treatment significantly inhibited the expression of EAU activity. In vivo and in vitro experiments revealed that this inhibitory effect was mediated by suppressing both Th1 and Th17 cells and through an up-regulation of IL-10 expression. These results suggest that IFN-β provides a potential treatment for diseases mediated by both Th1 and Th17 cells.

## Materials and Methods

### Reagents and Mice

Murine IL-12, IL-6, IL-23, anti-IFN-γ antibody and human TGF-β1 were purchased from R&D systems (Minneapolis, Minn, USA). Murine IFN-β was obtained from PBL (Piscataway, NJ, USA). Anti-CD3 and anti-CD28 antibodies were purchased from eBioscience (San Diego, CA, USA). IRBP_161-180_ (SGIPYIISYLHPGNTILHVD) was synthesized by Shanghai Sangon Biological Engineering Technology & Services Limited Company. LPS and CFA containing 1.0mg/ml of Mycobacterium tuberculosis was obtained from Sigma-Aldrich (St. Louis, MO).

B10RIII mice (6–8 weeks) were purchased from Jackson Laboratory and were housed under specific pathogen free conditions. All animals were treated according to the ARVO Statement for the Use of Animals in Ophthalmic and Vision Research.

### EAU induction and treatment

B10RIII mice (8–12 weeks) were immunized subcutaneously with a 200 µl emulsion containing 50 µg IRBP_161-180_ in CFA. Control mice received a subcutaneous injection of an emulsion of 50 µl PBS and 150 µl CFA. The clinical and histological scoring of EAU was performed according to criteria described previously [44]–[45]. Briefly, the severity of EAU was evaluated in a masked fashion of a scale of 0–4 based on the number, type, and size of lesions.

Murine IFN-β (1000U suspended in 100 µl of PBS) or PBS was administered subcutaneously to IRBP_161-180_ immunized mice every other day from day one before immunization to the end of the study. The mice were observed daily by slit lamp microscopy and ophthalmoscopy. On day 16 after immunization, the splenocytes or DLN cells (2×10^6^/ml) were stimulated with soluble anti-CD3 (1 µg/ml) and soluble anti-CD28 (1 µg/ml) or IRBP_161-180_(20 µg/ml) for 72 hours to detect the expression of IFN-γ, IL-17 and IL-10.

### Cell purification

CD4^+^CD62L^+^ naïve T cells were isolated from splenic single-cell suspensions with a CD4^+^CD62L^+^ T cell isolation kit (Miltenyi Biotec, Palo Alto, CA). Splenic single-cell suspensions were incubated with a cocktail of biotin-conjugated antibodies and anti-biotin microbeads for negative sorting of CD4^+^ T cells. CD4^+^ T cells were then used to separate CD4^+^CD62L^+/−^ T cells by addition of a recommended volume of anti-CD62L beads according to the manufacturer's instructions. The purity of isolated CD4^+^CD62L^+^ naïve T cells and CD4^+^CD62L^−^ effector/memory T cells, as identified by flow cytometry (FCM) analysis, was shown to be both higher than 95%. In addition, splenic single-cell suspensions were used to separate monocytes through incubation with the recommended volume of anti-CD11b beads according to the manufacturer's instructions. The purity of isolated CD11b^+^ monocytes, identified by FCM, was more than 95%.

### Cell culture

Splenocytes (2×10^6^/ml), obtained from EAU mice without IFN-β or PBS injection on days 0, 13, 16, 21, 28 and 35 after immunization, were stimulated with LPS (100ng/ml) for 72 hours and the supernatants were collected to evaluate the expression of IFN-β. On day 13 after immunization, splenocytes (2×10^6^/ml) obtained from EAU mice or control mice were stimulated with soluble anti-CD3 (1 µg/ml) and soluble anti-CD28 (1 µg/ml) or IRBP_161-180_ (20 µg/ml) for 72 hours and the supernatants were collected to examine IFN-γ and IL-17 expression.

CD4^+^CD62L^+^ naïve T cells at a concentration of 1.5×10^6^/ml, cultured in RPMI 1640 (Gibco BRL; Invitrogen) supplemented with 2% penicillin/streptomycin and 10% fetal bovine serum (complete medium) in 96-well plates were stimulated with soluble anti-CD3 and anti-CD28 (1 µg/ml) for 96 hours in Th1-polarizing(10ng/ml IL-12) or Th17-polarizing (20ng/ml IL-6, 5ng/ml TGF-β1 and 10ng/ml IL-23 with or without 2 µg/ml anti-IFN-γ) conditions in the presence or absence of IFN-β (250U/ml). CD4^+^CD62L^−^ (effector/memory) T cells at a concentration of 1.5×10^6^/ml were cultured for 72 hours in the presence or absence of IFN-β (250U/ml). Eventually, supernatants were collected to detect the expression of IFN-γ and IL-17.

### Coculture of IFN-β treated monocytes with CD4^+^CD62L^+/−^ T cells

Monocytes were stimulated with IFN-β (0–1000U/ml) for 24 hours. They were washed and cultured with CD4^+^CD62L^+^ (naïve) T cells or CD4^+^CD62L^−^ (effector/memory) T cells at a ratio of 1:5 in the presence of soluble anti-CD3 and anti-CD28 (1 µg/ml). Supernatants on day 3 were analyzed for IFN-γ and IL-17. Meanwhile, T cell proliferation was detected. IFN-β-treated monocytes were stimulated with LPS for another 24 hours and the supernatants were collected to analyze the levels of IL-6, TGF-β, IL-23 and IL-10.

### Cell proliferation

T cell proliferation was determined using a non radioactive colorimetric assay. 10 µl WST-8 (2-(2-methoxy-4-nitrophenyl)-3-(4-nitrophenyl)-5-(2, 4-disulfophen-yl)-2H-tetrazolium, monosodium salt) in 200 µl complete culture medium was added and incubated for 4 hours. The absorbance was determined at 450 nm using an ELISA reader (BIO-TEK Instruments).

### ELISA

Levels of IL-17, IFN-γ, IL-6, TGF-β, IL-23, IL-10 and IFN-β were measured using commercially available ELISA kits according to the manufacturer's instructions with detection limits of 15.6 pg/ml, 31.3 pg/ml, 15.6 pg/ml, 15.6 pg/ml, 31.3 pg/ml, 31.3 pg/ml and 15.6 pg/ml respectively. The following ELISA kits were purchased as indicated: IL-17, IFN-γ, IL-6, TGF-β and IL-10 (R&D Systems (Minneapolis, Minn, USA)); IL-23 (Bendermed system (Vienna, Austria, Europe)); IFN-β (PBL company (Piscataway, NJ, USA)).

### Statistical analysis

Statistical significance was analyzed by the one-way analysis of variance, Student *t* test and Mann-Whitney U test. Data were expressed as the mean ± standard deviation (SD). A *P* value below 0.05 was taken as a statistically significant difference.
