# Correction: Regulatory Effects of IFN-β on the Development of Experimental Autoimmune Uveoretinitis in B10RIII Mice

**DOI:** 10.1371/annotation/5934536e-ca5d-4ee8-bd71-7cfdc2e6d36c

**Published:** 2011-10-25

**Authors:** Min Sun, Yan Yang, Peizeng Yang, Bo Lei, Liping Du, Aize Kijlstra

The authors wish to add the following sentence to their Funding information: “This study was also supported by the National Natural Science Foundation of China (Grant No.81070722) http://159.226.244.22/portal/proj_search.asp."

